# Multi-target effects and clinical optimization treatment strategies of recombinant human B-type natriuretic peptide in acute heart failure and myocardial infarction

**DOI:** 10.3389/fphar.2026.1948386

**Published:** 2026-10-21

**Authors:** Yaling Zhou, Qiliang Hou, Yi Yang, Mengxiang Zhang, Zhongmou Zhang, Zhimin Dou, Bin Li

**Affiliations:** 1 The First School of Clinical Medicine, Lanzhou University and The First Hospital of Lanzhou University, Lanzhou, China; 2 Department of Critical Care Medicine, The First Hospital of Lanzhou University, Lanzhou, China

**Keywords:** acute heart failure, acute myocardial infarction, clinical optimization strategy, multi-target effect, recombinant human B-type natriuretic peptide, rhBNP

## Abstract

Acute cardiovascular diseases have become a serious public health challenge worldwide. Optimizing treatment strategies is crucial for improving the prognosis of patients. Recombinant human B-type natriuretic peptide (rhBNP) has garnered significant attention due to its multi-target mechanisms of action. This article aims to comprehensively review the multi-target effects of rhBNP in the treatment of acute cardiovascular diseases, and briefly explain its molecular mechanism through the activation of natriuretic peptide receptors and multiple downstream signaling pathways. In addition, combined with the latest clinical evidence, the optimization strategies for the clinical application of rhBNP are deeply discussed. However, it is important to acknowledge that current clinical evidence regarding renal protection and long-term clinical outcomes remains mixed. This review aims to provide evidence-based, individualized treatment recommendations for clinical practice, thereby improving the clinical outcomes of patients with acute cardiovascular diseases.

## 1 Introduction

Acute heart failure (AHF) and acute myocardial infarction (AMI) are the most common acute and critical illnesses in the field of cardiovascular medicine (Bozkurt et al., 2021), characterized by high incidence, high mortality and high rehospitalization rate (McDonagh et al., 2021; Heidenreich et al., 2022). According to the 2021 Global Burden of Disease Study, the number of heart failure patients worldwide has reached 55.5 million and is continuing to rise (Ran et al., 2025), and its 5-year survival rate is comparable to that of malignant tumors (Tsao et al., 2023). The 30-day mortality rate of acute ST-segment elevation myocardial infarction (STEMI) has decreased somewhat with modern treatments, but it still remains at 8%–15% (Byrne et al., 2023). At present, the treatment of heart failure mainly relies on diuretics to relieve the volume load, vasodilators to reduce the cardiac load, and positive inotropic drugs to increase cardiac output; while for myocardial infarction, reperfusion therapy is the core treatment approach. However, each of these treatment methods has its limitations. Diuretics can lead to deterioration of renal function, vasodilators are prone to developing resistance, positive inotropic drugs increase the risk of myocardial oxygen consumption and arrhythmias, and reperfusion itself can also induce cardiomyocyte apoptosis and microcirculation disorders (Qi et al., 2026). It is worth noting that heart failure and renal dysfunction often coexist and are mutually causal, forming cardiorenal syndrome. A decrease in cardiac output can lead to insufficient renal perfusion, and venous congestion can increase the pressure in the renal interstitium. Approximately 40%–50% of patients with acute decompensated heart failure have varying degrees of renal dysfunction (Heywood et al., 2007). Moreover, deterioration of renal function is an independent predictor of poor prognosis in heart failure (Forman et al., 2004). Therefore, exploring new drugs that can not only improve cardiac function but also alleviate renal function damage and have multiple protective effects has become a research hotspot in this field.

B- type natriuretic peptide (BNP) is a naturally occurring polypeptide hormone synthesized and secreted by ventricular muscle cells (Sudoh et al., 1988; Mukoyama et al., 1991). By binding to the natriuretic peptide receptor A (NPR-A), it activates the cyclic guanosine monophosphate -protein kinase G (cGMP-PKG) signaling pathway (Kuhn, 2004), resulting in vasodilation, diuresis and sodium excretion, inhibition of the renin-angiotensin-aldosterone system (RAAS system) and sympathetic nervous system, and other effects. It is an important compensatory mechanism for the body to respond to volume and pressure overload. However, in a severe state of heart failure, the compensatory effect of endogenous BNP is insufficient to completely counteract the over-activated vasoconstrictive system. Recombinant human B-type natriuretic peptide (rhBNP) is a BNP analogue artificially synthesized through genetic recombination technology, and its amino acid sequence, spatial structure and biological activity are exactly the same as those of endogenous BNP. Since the US Food and Drug Administration approved rhBNP for the treatment of acute decompensated heart failure in 2001, it has accumulated extensive clinical application experience worldwide and has been included in the Chinese guidelines for the diagnosis and treatment of acute heart failure.

While previous literature has discussed the general efficacy of rhBNP, a gap remains in linking its underlying molecular mechanisms with targeted clinical decision-making. To address this, this narrative review bridges key signaling pathways with individualized treatment optimization. Specifically, we summarize rhBNP’s multi-target cardiovascular mechanisms and evaluate practical strategies, including dose adjustment, combination therapy, baseline BNP-guided usage, and special population management, to provide actionable guidance for clinical practice.

## 2 Literature search strategy

To ensure a comprehensive review of the relevant literature, a structured search strategy was conducted. We searched major electronic databases, including PubMed, Embase, Cochrane Library, and Web of Science, for articles published from database inception up to July 2026. The search terms included a combination of Medical Subject Headings (MeSH) and free-text words: (“recombinant human B-type natriuretic peptide” OR “rhBNP” OR “nesiritide”) AND (“acute heart failure” OR “acute myocardial infarction” OR “acute cardiovascular diseases”). There were no strict language restrictions, though the focus was primarily on literature published in English and Chinese. The screening process involved evaluating the titles and abstracts of the retrieved records, followed by a full-text review of relevant articles. Studies were included if they investigated the pharmacological mechanisms, clinical efficacy, hemodynamic effects, safety, or optimization strategies of rhBNP in acute cardiovascular diseases.

## 3 The multi-target effects of rhBNP in acute cardiovascular diseases

Before detailing the specific pharmacological effects of rhBNP, it is essential to distinguish between surrogate or mechanistic outcomes and patient-centered clinical outcomes. Throughout this section, several studies report short-term improvements in hemodynamic parameters (e.g., PCWP, cardiac index), cardiac and renal biomarkers (e.g., NT-proBNP, CK-MB, troponin, cystatin-C, urine output), and inflammatory or oxidative-stress markers. While these findings are highly informative for understanding the biological mechanisms and physiological benefits of rhBNP, they must be interpreted with caution. These short-term physiological improvements should not be immediately interpreted as confirmed long-term clinical benefits, such as improved survival, reduced rehospitalization, prevention of dialysis, or enhanced functional status and quality of life.

### 3.1 The hemodynamic effect of rhBNP

Hemodynamic disorders are the core pathophysiological changes in both acute heart failure (AHF) and acute myocardial infarction (AMI), mainly manifested as increased cardiac preload and afterload, decreased cardiac output, and insufficient tissue perfusion. Traditional vasodilators (such as nitroglycerin) often exhibit resistance and reflex tachycardia. rhBNP, by mimicking the physiological effects of endogenous BNP, evenly dilates arteries, veins, and coronary arteries, improving the hemodynamics of patients and having the characteristics of rapid onset and long-lasting effects.

#### 3.1.1 Dilation of the arterial system and reduction of afterload

The renin-angiotensin-aldosterone system (RAAS) and the sympathetic nervous system in patients with heart failure are often in an overactive state, resulting in peripheral blood vessel constriction and a significant increase in the resistance of cardiac ejection. This increase in afterload further aggravates the metabolic demands of the already damaged heart, forming a vicious cycle. Therefore, reducing peripheral vascular resistance and alleviating the cardiac afterload are among the core goals of treating heart failure. rhBNP binds to NPR-A on vascular smooth muscle cells, activating the intracellular cGMP-PKG signaling pathway (Potter et al., 2009), causing the relaxation of vascular smooth muscle and thereby reducing systemic vascular resistance (Kuhn, 2004). In a randomized controlled trial of 42 patients with acute myocardial infarction complicated by heart failure, Li et al. evaluated IV rhBNP (1.5 μg/kg bolus, then 0.0075–0.03 μg/kg/min for 24 h; n = 21) *versus* nitroglycerin (10–100 μg/min; n = 21) with 30-h hemodynamic monitoring and a 1-week clinical follow-up. They found that after treatment with rhBNP, the pulmonary capillary wedge pressure (PCWP) of the patients decreased by 48.9% compared to the baseline (*P* < 0.05), and the cardiac index (CI) increased by 27.1% (*P* < 0.05) (Li et al., 2006). This effect can be observed 30 min after administration and persists until 6 h after discontinuation of the medication. The rapid and long-lasting effect of rhBNP in dilating arteries can quickly stabilize the patient’s hemodynamic state, providing time for subsequent treatment. Compared with the traditional vasodilator nitroglycerin, rhBNP has a more significant effect in dilating the arterial system (Publica tion Committee for the VMAC Investigators Vasodilatation in the Management of Acute CHF, 2002). Nitroglycerin mainly dilates the venous system, so its effect in reducing afterload is limited. However, rhBNP can simultaneously dilate both arteries and veins, effectively reducing both the preload of the heart and the afterload, achieving a more comprehensive hemodynamic optimization. This “balanced dilation” characteristic is an important advantage of rhBNP compared to traditional vasodilators. However, it should be noted that while the rapid optimization of PCWP and CI represents a significant physiological improvement in the acute phase, these hemodynamic changes are surrogate endpoints. Further evidence is required to definitively determine whether such short-term hemodynamic stabilization translates into long-term patient-centered benefits, such as reduced mortality or lower heart failure rehospitalization rates.

#### 3.1.2 Dilation of the venous system and reduction of preload

Patients with heart failure often experience an increase in venous return due to the compensatory contraction of the venous system, resulting in blood accumulation in the pulmonary circulation and exacerbating symptoms of pulmonary edema and breathing difficulties (Boorsma et al., 2020). Therefore, reducing preload is the key to alleviating pulmonary edema (Funk et al., 2013). The venous return volume (VR) is determined by the mean systemic filling pressure (MSFP), the right atrial pressure (CVP), and the venous return resistance (RVR). The calculation formula is VR = (MSFP - CVP)/RVR. Traditional vasodilators (such as nitroglycerin) mainly reduce venous return flow by lowering MSFP, thereby reducing preload. However, excessive reduction of venous return flow may lead to a decrease in cardiac output. The mechanism of action of rhBNP is different: it dilates the venous capacity vessels, reduces the resistance of venous return, and while reducing the preload of the heart, maintains the venous return volume. In a retrospective study of patients with acute decompensated heart failure (ADHF) applying Guytonian mathematical modeling to clinical hemodynamic data, Liu et al. reported that in patients with acute decompensated heart failure (ADHF), the infusion of rhBNP 3 h later led to a significant decrease in RVR and MSFP, with a slight increase in CVP (all *P* < 0.05) (Liu et al., 2021). Although the pressure gradient driving venous return was reduced, due to the greater decrease in RVR, cardiac output was maintained. Therefore, the effect of rhBNP on the venous system is not to increase the pressure gradient of venous return, but to improve the efficiency of venous return by reducing the resistance of venous return. From a clinical perspective, the effect of rhBNP in reducing preload is directly manifested as a rapid decrease in PCWP and a rapid relief of respiratory distress symptoms. rhBNP expands the venous system and “stores” blood in peripheral veins, thereby alleviating pulmonary circulation congestion. Since the venous return volume is maintained, the cardiac output does not significantly decrease due to the reduction in preload, which makes rhBNP have unique clinical value in the treatment of acute heart failure. However, because these conclusions are based on mathematical calculations and physiological modeling from observational data, statements regarding the preservation of venous return and cardiac output should be interpreted with caution, and direct prospective clinical measurements remain necessary to confirm these effects.

#### 3.1.3 Dilation of the coronary arteries and increased myocardial perfusion

The core issue of myocardial ischemia is the imbalance between oxygen supply and demand. In patients with heart failure and myocardial infarction, the coronary artery perfusion pressure decreases, and the myocardial oxygen consumption increases, further aggravating myocardial damage. rhBNP activates the NPR-A receptor on the smooth muscle cells of the coronary artery, causing the smooth muscle to relax, dilating the epicardial coronary artery and coronary resistance vessels, thereby increasing myocardial oxygen supply and reducing myocardial oxygen consumption (Zellner et al., 1999). In a cardiac catheterization laboratory setting involving 10 patients (mean age 58 ± 12 years; 5 with CAD and five without), Michaels et al. evaluated intravenous nesiritide using quantitative coronary angiography (QCA) for vessel lumen diameter, an intracoronary Doppler guidewire for coronary blood flow, and coronary sinus catheterization for myocardial oxygen consumption, it was shown that 30 min after rhBNP infusion, the diameter of the epicardial coronary artery increased by 15% compared to the baseline (*P* = 0.007), coronary blood flow increased by 35% (*P* = 0.031), while myocardial oxygen consumption decreased by 8% (*P* = 0.043) (Michaels et al., 2003). The regulatory effect of rhBNP on myocardial metabolism is in sharp contrast to that of positive inotropic drugs. Positive inotropic drugs such as dobutamine, although they increase cardiac output, also significantly increase myocardial oxygen consumption, which may induce arrhythmias and myocardial ischemia. However, rhBNP increases cardiac output while reducing myocardial oxygen consumption without increasing heart rate. This characteristic of maintaining an oxygen supply-demand balance makes it more advantageous for application in patients with myocardial ischemia. However, clinical selection between rhBNP and positive inotropes ultimately depends on patient blood pressure, tissue perfusion, and the presence or absence of cardiogenic shock. In severe hypotension or cardiogenic shock, positive inotropes remain essential to restore systemic perfusion, whereas rhBNP provides significant advantages in normotensive or hypertensive acute heart failure without cardiogenic shock by reducing cardiac load without raising oxygen demand.

### 3.2 The myocardial protective effect of rhBNP

After acute myocardial infarction, emergency percutaneous coronary intervention (PCI) can rapidly restore blood flow in the infarct-related arteries, which is currently the most effective reperfusion treatment method. However, reperfusion itself can induce additional myocardial damage, known as “ischemia-reperfusion (IR) injury” (Yellon and Hausenloy, 2007). IR injury can lead to cardiomyocyte apoptosis, microcirculation disorders and ventricular remodeling, thereby negating some of the reperfusion benefits and seriously affecting the prognosis of patients. Therefore, while restoring blood flow, protecting cardiomyocytes from reperfusion injury is the key to improving the prognosis of AMI. rhBNP not only has hemodynamic effects but also exerts myocardial protective effects by preventing ischemia-reperfusion injury, reducing the myocardial infarction area and inhibiting ventricular remodeling.

#### 3.2.1 Reduce the area of myocardial infarction

After acute myocardial infarction, the size of the myocardial infarction area is a key factor determining the long-term prognosis of patients. The larger the infarction area, the more severe the deterioration of cardiac function, and the higher the risks of heart failure and death. Therefore, further reducing the size of the myocardial infarction area on the basis of reperfusion therapy is an important strategy for improving the prognosis of AMI. Clinical studies have confirmed that rhBNP can effectively reduce the area of myocardial infarction. In a randomized controlled trial by Li et al. (N = 106 patients with acute anterior STEMI undergoing primary PCI, randomized 1:1 via a random number table), patients received post-ischemic conditioning (PC) with or without an rhBNP regimen (a 1.5 μg/kg IV bolus before PCI followed by 0.0075 μg/kg/min continuous infusion for 72 h). Infarct size was indirectly assessed using the 72-h area under the curve (AUC) for cardiac enzymes (CK-MB and TnI) and post-procedural Wall Motion Score Index (WMSI). The rhBNP group demonstrated a 27% reduction in CK-MB AUC and an 18% reduction in TnI AUC compared to PC alone (*P* < 0.05). This result indicates that, in addition to mechanical myocardial protection measures, rhBNP can provide additional and cumulative myocardial protective effects. However, reductions in CK-MB and TnI serve as surrogate markers for infarct size and still require validation against long-term survival or functional outcomes. Further analysis revealed that the corrected TIMI blood flow frames (cTFC) in the rhBNP group was significantly lower than that in the control group (21.0 vs. 28.2, *P* = 0.005), while there was no statistically significant difference in the TIMI grade 3 blood flow and myocardial color grading (MBG) between the two groups (Li et al., 2015). cTFC is a more sensitive indicator reflecting microcirculation function. This result suggests that the improvement of myocardial perfusion by rhBNP mainly occurs at the microcirculation level, rather than in the epicardial large blood vessels.

#### 3.2.2 Inhibit ventricular remodeling

Ventricular remodeling is a critical contributing factor to the deterioration of cardiac function following acute myocardial infarction (AMI), with its hallmark features encompassing cardiomyocyte hypertrophy, interstitial fibrosis, extracellular matrix remodeling, and ventricular chamber dilation (Cohn et al., 2000; Pfeffer and Braunwald, 1990). This remodeling process is primarily driven by three distinct factors: a larger initial infarct size correlates with more pronounced remodeling; a more severe post-reperfusion inflammatory response leads to aggravated fibrosis; and a higher hemodynamic load accelerates ventricular dilation (Frantz et al., 2022). Notably, rhBNP exerts targeted interventions against all three of these driving factors (Hayashi et al., 2001). A study in 2015 showed that the wall motion score index (WMSI) of the rhBNP group at 1 week after surgery was significantly better than that of the control group (1.74 vs. 2.40, *P* < 0.001), indicating that local wall motion function had begun to recover (Li et al., 2015). The wall motion score index is an important indicator for evaluating the left ventricular systolic synchrony and local function. Its improvement reflects the increase in viable myocardial mass and the recovery of systolic function. Furthermore, it only reflects the early functional improvement, rather than the ultimate preventive effect on long-term ventricular remodeling.

### 3.3 Renal effects and potential renal protection of rhBNP

Heart failure is often accompanied by renal dysfunction, resulting in “Cardiorenal syndrome” (Rangaswami et al., 2019; Ndumele et al., 2023; Jefferies et al., 2023). During heart failure, the patient’s cardiac output decreases, leading to insufficient renal perfusion, which subsequently activates the RAAS and sympathetic nervous system, causing renal vessels to constrict, thus entering a vicious cycle of heart failure and renal failure. Moreover, the venous congestion caused by heart failure can increase the pressure in the renal interstitium, reducing the glomerular filtration rate. Additionally, the diuretics commonly used by heart failure patients can exacerbate kidney damage, and the contrast agents used in PCI procedures may cause contrast-induced nephropathy (CIN). Approximately 40%–50% of patients with acute decompensated heart failure have varying degrees of renal dysfunction, and renal function deterioration is an independent predictor of poor prognosis in right ventricular heart failure. Therefore, improving cardiac function while protecting the kidneys is an important goal in the treatment of heart failure. rhBNP has multiple effects such as vasodilation, diuresis and sodium excretion, and renin-angiotensin-aldosterone system inhibition, which may have a beneficial impact on renal function.

#### 3.3.1 The effect of rhBNP on the basic renal function

Renal hypoperfusion caused by heart failure is one of the core mechanisms of renal function impairment. The effect of rhBNP on renal circulation is not simply to dilate renal vessels and increase total blood flow, but rather to redistribute blood flow within the kidneys after dilating the vessels to improve renal perfusion (Jensen et al., 1998). Uri Elkayam et al. used intravascular Doppler and quantitative angiography to assess renal hemodynamics. The study found that 15 min after the administration of rhBNP, the diameter of the renal artery increased by 8% compared to the baseline (*P* < 0.001), but there was no significant change in renal blood flow (Elkayam et al., 2008). This result suggests that the main effect of rhBNP is to dilate the renal arteries and reduce renal vascular resistance, thereby optimizing the blood flow distribution within the kidneys and improving the ischemic state of the kidneys.

Venous congestion caused by heart failure can lead to an increase in interstitial pressure in the kidneys, further damaging kidney function. The diuretic effect of rhBNP can alleviate venous congestion, lower interstitial pressure in the kidneys, and further promote the early recovery of kidney function. A study published in 2015 indicated that the total urine volume of the rhBNP group was significantly higher than that of the control group within 72 h (*P* < 0.001) (Xing et al., 2016). A more sensitive marker of renal function, cystatin-C, also confirmed this effect: the cystatin-C level in the rhBNP group was significantly lower than that in the control group at 24 h (*P* = 0.034) and 72 h (*P* = 0.049), while there were no significant differences in traditional indicators such as serum creatinine and eGFR between the two groups. Similarly, increased urine output and cystatin-C lowering represent short-term physiological responses rather than confirmed prevention of long-term dialysis or renal failure. In addition, rhBNP can also exert its effects by antagonizing the RAAS and inhibiting the sympathetic nervous system, thereby reducing renal vascular contraction. A study combining rhBNP with ivabradine indicated that the levels of ALD, NE, and AngII in the combined treatment group were significantly lower than those in the group using ivabradine alone (*P* < 0.001) (Zhang et al., 2026). This neuroendocrine regulation can further improve renal perfusion and protect renal function.

#### 3.3.2 rhBNP for the prevention of contrast-induced nephropathy (CIN) following PCI

Patients with heart failure have a significantly increased risk of contrast-induced nephropathy due to factors such as inadequate renal perfusion and fluid restriction (Mehran et al., 2019; Zhang et al., 2022; Liu et al., 2016; Carullo et al., 2026), with an incidence rate as high as 24.6%–29.7% in STEMI patients with concurrent heart failure (Zhang et al., 2010a; Zhang et al., 2010b). rhBNP mitigates the risk of CIN through the following mechanisms: First, the diuretic effect of rhBNP accelerates the renal clearance of contrast media. As previously described, rhBNP significantly increases urine output, thereby reducing the retention time and concentration of contrast agents within the renal tubules; this diminishes their direct cytotoxicity and ultimately prevents the development of contrast-induced nephropathy. Second, rhBNP improves intrarenal hemodynamics. Although it does not alter total renal blood flow, rhBNP dilates the renal arteries and redistributes intrarenal blood flow. A study has clearly demonstrated that the incidence of CIN in the rhBNP group was significantly lower than that in the control group (13.5% vs. 24.0%, *P* < 0.05), with a relative risk reduction of approximately 44% (Zhang et al., 2010a). Another study result also indicated that the incidence of CIN in the rhBNP group was lower than that in the control group (12.28% vs. 28.81%, *P* = 0.049), and through multivariate analysis, rhBNP was identified as one of the independent protective factors for CIN (*OR* = 0.202) (Xing et al., 2016).

#### 3.3.3 Safety of rhBNP in heart failure patients with renal insufficiency

Current studies have not found that rhBNP has a definite renal toxicity. The risk of acute renal failure (ARF) mainly depends on the patient’s baseline characteristics rather than the drug itself. The results of a study by Jose Iglesias et al. showed that the independent predictors of AFR were the levels of BUN and BNP at admission, and rhBNP did not independently increase the risk of AFR (*P* = 0.42, *OR* = 0.83). The predictive factors for the rhBNP group pointed to insufficient renal perfusion and abnormal renal autoregulation, while the predictive factors for the non-rhBNP group pointed to reduced basic renal function and age (Iglesias et al., 2006). Therefore, for patients with the above high-risk characteristics, renal function monitoring should be strengthened when using rhBNP.

### 3.4 The anti-inflammatory and antioxidative roles of rhBNP

During the pathophysiological processes of heart failure and acute myocardial infarction, there is a continuous inflammatory response and oxidative stress (Tsutsui et al., 2011). Inflammatory factors such as TNF-α and IL-6 can inhibit myocardial contractility and promote ventricular remodeling (Feldman et al., 2000); while oxidative stress generates a large amount of oxygen free radicals, which damage myocardial cells. Preliminary clinical evidence suggests that rhBNP may help reduce the levels of serum inflammatory factors (TNF-α, IL-6, CRP/hs-CRP) and oxidative stress indicators (MDA, XO), while potentially enhancing the activity of antioxidant enzymes (SOD, GPx). Some observations indicate that this anti-inflammatory effect may occur early in the treatment course. For instance, a small-scale study on patients with acute heart failure showed that after 1 day of treatment, the levels of TNF-α, IL-6, and CRP in the serum of patients in the rhBNP group were significantly lower than those in the control group (*P* < 0.05) (Luo et al., 2024). Furthermore, a small prospective randomized controlled trial (RCT) reported that after 1 week of treatment, the GSH-PX level in the rhBNP group was significantly higher (*P* = 0.01) and the MDA level was significantly lower (*P* = 0.001) than in the control group (Pang et al., 2021). In another study targeting patients with severe heart failure, the rhBNP combined with tolvaptan group showed significantly lower levels of inflammatory factors such as TNF-α and IL-33 after treatment compared to the tolvaptan monotherapy group (Yang et al., 2023). However, these findings must be interpreted with caution. Reductions in serum inflammatory markers (e.g., TNF-α, IL-6) and oxidative stress biomarkers (e.g., ROS, MDA) should be viewed as potential biological effects rather than confirmed clinical endpoints. Importantly, whether these biochemical improvements translate into clinically meaningful cardiovascular outcomes, such as reduced all-cause mortality, lower heart failure rehospitalization, or long-term functional recovery, remains unproven and requires further robust investigation. Most of these clinical studies involve combination therapies, making it difficult to definitively determine whether the observed anti-inflammatory and antioxidant effects are driven by rhBNP alone or by the synergistic effect of the combined medications. Furthermore, current evidence is largely drawn from studies with relatively small sample sizes, and there is a lack of high-quality, large-scale RCTs evaluating rhBNP as a monotherapy against a placebo control.

## 4 Molecular mechanisms and signaling pathways underlying the multi-target effects of rhBNP

The multi-target effects of rhBNP are mediated through its specific binding to the NPR-A receptor and subsequent activation of downstream signaling cascades. It should be emphasized that while the hemodynamic actions of rhBNP are well-documented in human clinical studies, much of our current understanding regarding its specific downstream protective pathways, particularly anti-inflammatory, antioxidant, and direct anti-apoptotic signaling, is derived from preclinical experimental systems (including *in vitro* cell cultures, non-cardiac tissue models, and animal models of acute injury). These preclinical findings provide a supportive biological rationale rather than established clinical effects in human cardiovascular diseases.

### 4.1 The core NPR-A/cGMP/PKG signaling pathway

The NPR-A/cGMP/PKG pathway is the most fundamental and upstream signaling pathway for the action of rhBNP, and it serves as the basis for all other pathways. rhBNP binds specifically to the NPR-A on the cell membrane, thereby triggering the core signal transduction mechanism. NPR-A, also designated as Guanylate Cyclase A (GC-A), has a guanylate cyclase (pGC) domain in its intracellular segment. After rhBNP binds to the receptor, it activates the PGC domain, catalyzing the conversion of guanosine triphosphate (GTP) into the second messenger cyclic guanosine monophosphate (cGMP). cGMP, as a key signaling molecule, activates protein kinase G (PKG), which in turn phosphorylates various downstream target proteins, generating a series of biological effects. In different cell types, the cGMP/PKG pathway can produce different effects. As demonstrated in experimental cellular and tissue models, the cGMP/PKG signaling cascade yields cell-type-specific responses: In vascular smooth muscle cells, experimental studies demonstrate that the cGMP/PKG pathway phosphorylates the core regulatory subunit of myosin light chain phosphatase (MLCP), promoting myosin dephosphorylation, while inhibiting L-type calcium channels and activating potassium channels, resulting in relaxation of vascular smooth muscle and dilation of blood vessels (Mishra et al., 2025; Numata and Takimoto, 2022). In cardiac muscle cells, *in vitro* and animal models suggest that the cGMP/PKG pathway inhibits calcium influx mediated by L-type calcium channels and reduces the sensitivity of myofilaments to calcium ions, thereby weakening myocardial contractility and reducing myocardial oxygen consumption. In the kidneys, preclinical experimental evidence indicates that the cGMP/PKG pathway reduces sodium reabsorption by inhibiting the Na+-K+-ATPase on the basolateral membrane of renal tubular epithelial cells and the sodium-hydrogen exchanger on the apical membrane. It promotes sodium excretion and diuretic effects. At the same time, this pathway can dilate the afferent arterioles, increase renal blood flow (Renal Blood Flow, RBF) and glomerular filtration rate (Glomerular Filtration Rate, GFR), thereby improving renal function. Therefore, the NPR-A/cGMP/PKG signaling pathway is the core molecular mechanism by which rhBNP exerts various pharmacological effects such as vasodilation, cardiac protection, and diuresis and sodium excretion (Figure 1).

**FIGURE 1:**
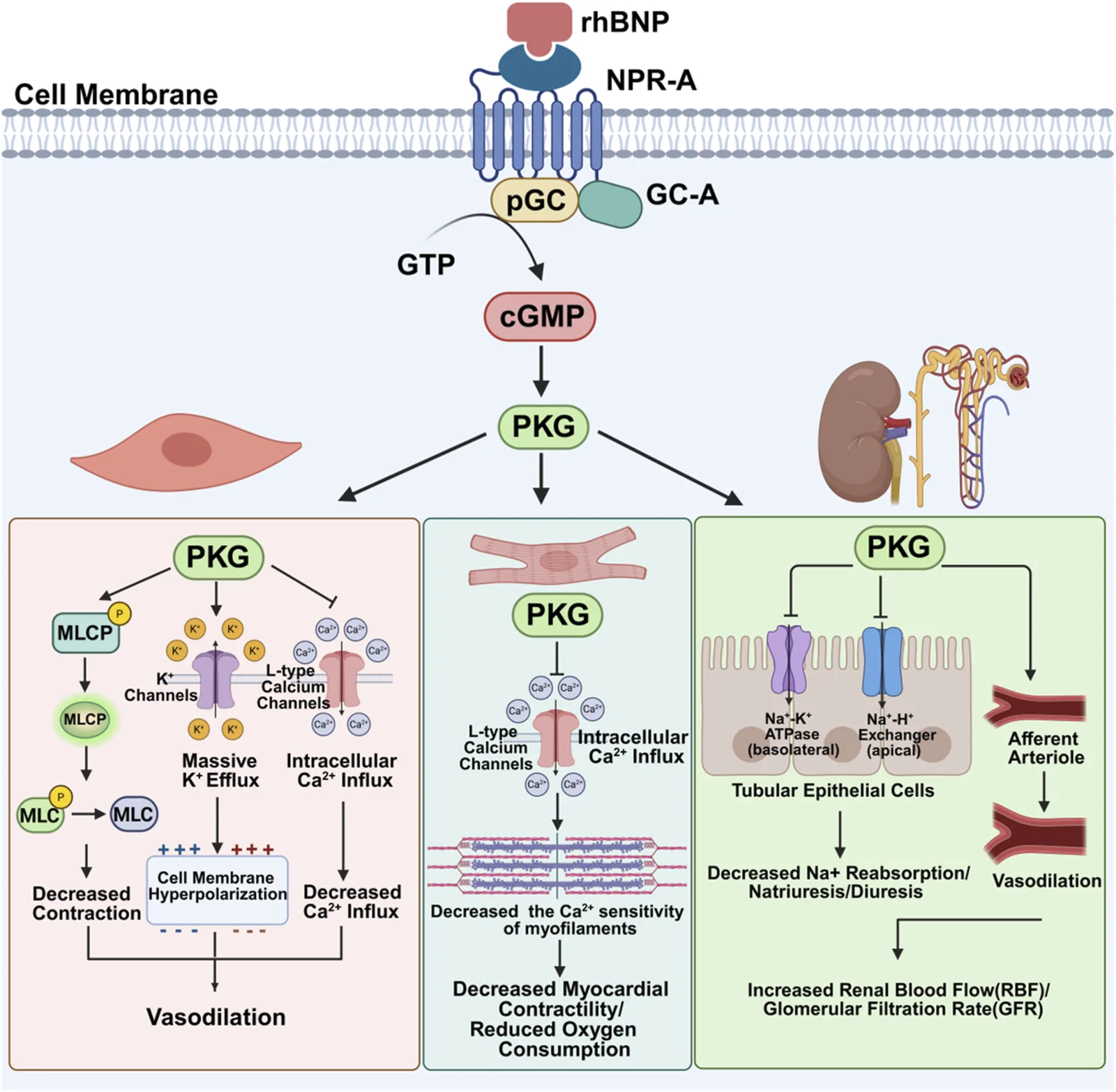
rhBNP binds to NPR-A to activate the cGMP/PKG signaling cascade, exerting tissue-specific effects: inducing vasodilation in vascular smooth muscle cells (left), reducing myocardial contractility and oxygen consumption in cardiomyocytes (middle), and promoting natriuresis, diuresis, and improved renal perfusion in renal tubular epithelial cells (right). rhBNP: Recombinant human B-type natriuretic peptide NPR-A: Natriuretic peptide receptor A cGMP: Cyclic guanosine monophosphate PKG: Protein kinase G MLCP: myosin light chain phosphatase MLC: Myosin Light Chain ↓: Activation / Promotion ⊥: Inhibition / Reduction.

### 4.2 Signal pathways for myocardial protection

The “terminal event” of reperfusion injury is the opening of the mitochondrial permeability transition pore (mPTP) (Hausenloy and Yellon, 2013). In the early stage of reperfusion, the accumulation of intracellular calcium and the burst of oxygen free radicals jointly trigger the opening of the mPTP, resulting in the collapse of mitochondrial membrane potential, impairment of ATP synthesis, release of cytochrome C, and ultimately activation of the Caspase-dependent apoptotic pathway (Heusch, 2020). Therefore, inhibiting the opening of mPTP is considered a key proposed target for myocardial protection (Halestrap, 2009). Evidence regarding rhBNP-mediated cardioprotection and anti-apoptotic pathways is predominantly derived from animal models of ischemia-reperfusion injury and isolated cardiomyocyte experiments. In these experimental models, rhBNP is proposed to contribute to myocardial protective effects through two main pathways: the mitochondrial ATP-sensitive potassium channel (mK-ATP) pathway and the reperfusion injury salvage kinase pathway (RISK) (Sun et al., 2009; Sun et al., 2010), both of which may act on the mPTP as a final common target. mK-ATP pathway: Located on the inner mitochondrial membrane, this channel may be activated by rhBNP via the NPR-A/cGMP/PKG signaling cascade. In experimental models, this activation promotes potassium ion influx into the mitochondria. The influx of potassium ions reduces the mitochondrial membrane potential difference, decreases the driving force for calcium ion influx, thereby alleviating mitochondrial calcium overload and inhibiting mPTP opening. RISK: It is the core mechanism of endogenous cardiac protection during the early reperfusion period, mainly including two branches: phosphatidylinositol 3-kinase (PI3K)/protein kinase B (also known as Akt) and extracellular signal-regulated kinase (Erk1/2). This pathway inhibits mPTP opening by phosphorylating downstream targets such as glycogen synthase kinase-3β (GSK-3β), thereby blocking the apoptotic signal (Hausenloy and Yellon, 2004; Yang et al., 2006). The above two pathways do not exist independently but operate in synergy. mK-ATP inhibits the opening of mPTP by reducing the mitochondrial membrane potential difference, while RISK blocks the apoptotic signal by phosphorylating downstream effect molecules. Together, they act on the final target of mPTP, providing more comprehensive protection for cardiomyocytes (Figure 2). However, it should be clarified that while these pathways have been identified in animal models and isolated cell studies, direct evidence confirming these specific signaling mechanisms in human myocardial tissue following rhBNP treatment is currently lacking.

**FIGURE 2:**
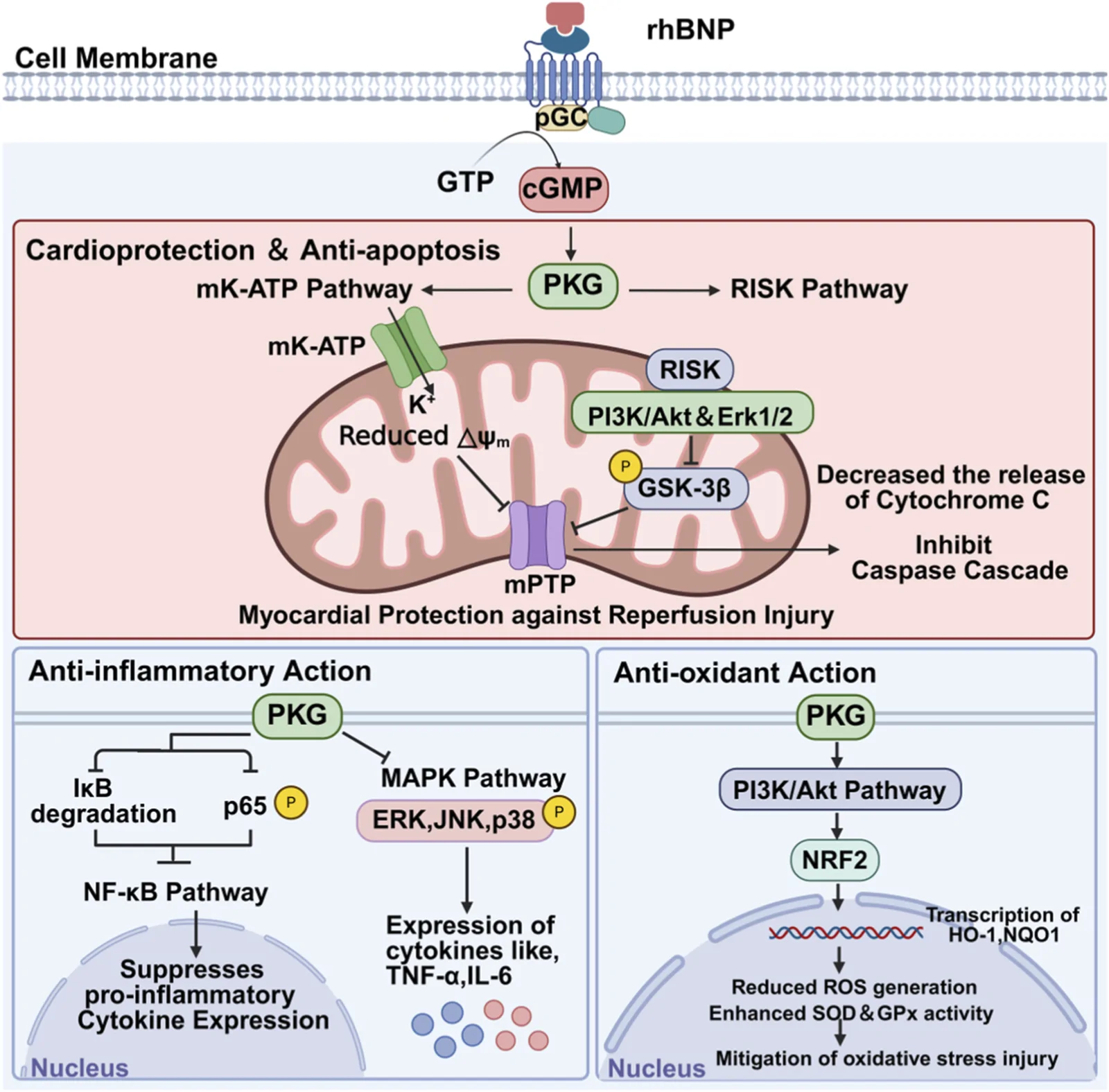
rhBNP/NPR-A signaling protects the heart by inhibiting mPTP opening via mK-ATP and RISK pathways. Furthermore, it suppresses inflammatory cytokine expression by inhibiting NF-κB and MAPK pathways, and mitigates oxidative stress by upregulating the NRF2/HO-1 signaling axis. rhBNP: Recombinant human B-type natriuretic peptide NPR-A: Natriuretic peptide receptor A cGMP: Cyclic guanosine monophosphate PKG: Protein kinase G mPTP: mitochondrial permeability transition pore RISK: reperfusion injury salvage kinase GSK-3β: glycogen synthase kinase-3β ↓: Activation / Promotion ⊥: Inhibition / Reduction.

### 4.3 Anti-inflammatory signaling pathways

It is important to emphasize that current evidence regarding the anti-inflammatory properties of rhBNP is largely derived from non-cardiac experimental systems, such as *in vitro* cell cultures and animal models of acute systemic or lung inflammation, rather than established clinical cardiovascular studies in humans. In these experimental models, rhBNP has been observed to modulate major inflammatory signaling pathways, including the NF-κB and MAPK pathways (Hayden and Ghosh, 2008; Lawrence, 2009). NF-κB pathway: NF-κB is the core transcription factor that regulates the transcription of inflammatory factors. In the resting state, NF-κB binds to the inhibitory protein IκB and exists in the cytoplasm, in an inactive state. When the cells are stimulated by inflammation, IκB is phosphorylated and degraded, releasing NF-κB and allowing it to translocate into the nucleus, initiating the transcription of inflammatory factors such as TNF-α and IL-6. rhBNP inhibits the phosphorylation of p65 and the degradation of IκB through the cGMP/PKG pathway, blocking the activation of the NF-κB pathway and thereby reducing the expression of pro-inflammatory factors. In the rat model of acute lung injury, rhBNP can inhibit the phosphorylation of p65 and the degradation of IκB, blocking the activation of the NF-κB pathway (Song et al., 2015). An *in vitro* macrophage experiment also found that rhBNP can dose-dependently inhibit the phosphorylation of p65 in the NF-κB pathway, thereby down-regulating the expression of IL-6 and TNF-α (Li et al., 2018). MAPK pathway: The MAPK family (ERK, JNK, P38) is another pathway involved in the transcriptional regulation of inflammatory factors (Kyriakis and Avruch, 2012). rhBNP can inhibit the phosphorylation of ERK, JNK and P38, thereby down-regulating the expression of IL-6 and TNF-α. It is notable that the inhibitory effect of rhBNP on P-ERK is relatively weak, suggesting that different MAPK subfamilies may have different sensitivities to rhBNP (Figure 2). Although derived from macrophage/inflammation models, these pathways are critical in AHF and AMI, where macrophage activation *via* NF-κB and MAPK directly drives post-infarct inflammatory damage, microvascular dysfunction, and adverse remodeling.

### 4.4 Antioxidant signaling pathways

Similar to its anti-inflammatory actions, the antioxidant potential of rhBNP is primarily supported by preclinical animal models and *in vitro* cellular studies. rhBNP exerts antioxidant effects through the PI3K/AKT/NRF2/HO-1 pathway. NRF2 is the core transcription factor that regulates the expression of antioxidant enzymes. In the resting state, NRF2 binds to Keap1 and exists in the cytoplasm. Under oxidative stress stimulation, NRF2 is released from Keap1 and translocates into the nucleus, initiating the antioxidant response element (ARE), and inducing the transcription of antioxidant enzyme genes such as HO-1 (heme oxygenase-1) and NQO1 (NAD(P)H quinone oxidoreductase 1). rhBNP upregulates the expression of NRF2 by activating the PI3K/AKT pathway, thereby inducing the expression of antioxidant enzymes such as HO-1 and NQO1. An animal experiment has shown that rhBNP can upregulate the expression of NRF2 and HO-1 by activating the PI3K/AKT pathway, achieving the purpose of inhibiting oxidative stress and preventing cell apoptosis (Li et al., 2020). Through these mechanisms, rhBNP can significantly reduce the generation of reactive oxygen species (ROS), while enhancing the activity of antioxidant enzymes such as superoxide dismutase (SOD) and glutathione peroxidase (GPx), thereby alleviating oxidative stress damage (Figure 2).

## 5 Clinical optimization treatment strategy for rhBNP

While preclinical models and short-term clinical observations demonstrate the multi-target and hemodynamic benefits of rhBNP, landmark large-scale randomized controlled trials, such as ASCEND-HF and ROSE-AHF, revealed neutral outcomes regarding 30-day all-cause mortality, rehospitalization, and renal protection (O’Connor et al., 2011; Wan et al., 2016). This discrepancy between short-term physiological improvements and long-term hard clinical endpoints highlights that a “one-size-fits-all” treatment paradigm is insufficient for heterogeneous acute cardiovascular populations. Consequently, the clinical application of rhBNP must transition from general efficacy validation toward precision optimization. To bridge this gap, the following sections comprehensively explore individualized treatment strategies, including precise dose titration, synergistic combination therapies, baseline biomarker-guided stratification, and tailored management for special populations, to maximize the therapeutic efficacy and safety profile of rhBNP.

### 5.1 Dose optimization strategy

The dose-response relationship of rhBNP indicates that while low doses are effective, high doses offer no additional clinical benefit and may increase the risk of adverse events. This phenomenon has been confirmed in multiple studies. A study on acute decompensated heart failure compared four administration regimens: the low-dose short-course group, the low-dose long-course group, the high-dose short-course group, and the high-dose long-course group. The results showed that the low-dose short-course group had the most significant improvement in systemic symptoms, while the high-dose long-course group had an advantage in the decrease of N-terminal pro-B-type natriuretic peptide (NT-proBNP), but also had the highest incidence of adverse events (Recombinant Human Brain Natriuretic Peptide Multicenter Clinical Study Group, 2011). This suggests that the balance point between the efficacy and safety of rhBNP may lean towards the low-dose regimen. When using rhBNP in clinical practice, dose selection must be handled with precision, distinguishing between the US FDA-approved nesiritide and the domestic rhBNP product utilized in China. While nesiritide trials (such as the VMAC trial) explored maintenance doses up to 0.03 μg/kg/min, Chinese clinical practice guidelines and expert consensus recommend a more conservative dosing strategy for domestic rhBNP (中华医学会心血管病学分会 and 中国医师协会心血管内科医师分会, 2024). Specifically, the standard recommended regimen starts with a continuous intravenous infusion of 0.0075 μg/kg/min to 0.01 μg/kg/min (often following an initial loading dose of 1.5–2 μg/kg/min, which may be omitted in hemodynamically sensitive patients) (中华医学会心血管病学分会 and 中国医师协会心血管内科医师分会, 2024). The infusion rate should be adjusted carefully based on continuous blood pressure monitoring and clinical responses. Aggressive up-titration is discouraged, as high doses offer no additional clinical benefit and significantly increase the risk of dose-dependent hypotension. For patients with combined renal dysfunction, since the clearance rate of rhBNP slows down, a lower initial dose (such as 0.005 μg/kg/min) should be selected, and the interval for dose adjustment should be appropriately extended to prevent hypotension and further deterioration of renal function (Adel et al., 2020). For the patient group with unstable hemodynamics, this individualized dosing strategy helps to strike a balance between efficacy and safety.

### 5.2 Combination therapy strategy

#### 5.2.1 Combined with positive inotropic drugs

In a small cohort of elderly patients with acute heart failure and hypotension (N = 213; Age > 60 years; Systolic blood pressure ≤ 110 mmHg or mean arterial pressure ≤ 65 mmHg), adding dobutamine or dopamine to rhBNP for 3 days significantly improved LVEF and decreased NT-proBNP compared to rhBNP alone (*P* < 0.05) (Meng et al., 2025). Short-term adverse events (e.g., severe hypotension or fatal arrhythmias) were similar between groups, though long-term hard endpoints (e.g., mortality, rehospitalization) were not evaluated (Meng et al., 2025). This combined treatment mechanism is complementary: positive inotropic drugs maintain blood pressure by increasing myocardial contractility, while rhBNP reduces cardiac load through diuresis and vasodilation. Together, they improve cardiac function and solve the problem that rhBNP may cause hypotension. For patients with heart failure who have low blood pressure or are intolerant to vasodilators, this combined treatment has significant clinical value. Crucially, inotropic agents should not be routinely combined with rhBNP; they are strictly indicated for selected patients with symptomatic hypotension or tissue hypoperfusion to restore perfusion, whereas normotensive or hypertensive AHF patients should receive rhBNP monotherapy or standard vasodilators.

#### 5.2.2 Combined with and sequential use of sacubitril/valsartan sodium

Sacubitril/Valsartan Sodium is an angiotensin receptor-neprilysin inhibitor (ARNI), which, while inhibiting the RAAS system, can enhance the activity of endogenous natriuretic peptides (Solo et al., 2017). A recent retrospective study evaluated patients with acute left heart failure and observed that after treatment with rhBNP combined with sacubitril/valsartan sodium, cardiac function, inflammatory markers, and oxidative stress indicators were significantly better than those in the sacubitril monotherapy group (Gao and Cheng, 2025). However, because this evidence is derived from a retrospective analysis, these findings are subject to inherent selection biases and unmeasured confounding factors; thus, the clinical superiority of this combination strategy requires further prospective validation before it can be considered an established standard. Both drugs act on the natriuretic peptide system, but their mechanisms of action are different. rhBNP directly replenishes exogenous BNP, while sacubitril/valsartan sodium inhibits neprilysin to reduce the degradation of endogenous BNP. The combination of the two can synergistically enhance efficacy. In a sequential treatment strategy, one study noted that a sequential treatment group maintained significantly better LVEF at 36 weeks compared to a standard treatment group and an rhBNP monotherapy group (*P* < 0.05) (Pang et al., 2021). This strategy aims to achieve rapid symptom relief in the acute phase while providing long-term maintenance treatment in the chronic phase, though larger robust trials are necessary to confirm these preliminary benefits.

#### 5.2.3 Combined with ivabradine

Ivabradine is a specific inhibitor of cardiac pacemaker current (If), which reduces myocardial oxygen consumption by lowering heart rate. In a prospective study of 126 patients with ischemic cardiomyopathy and heart failure (LVEF≤40%, NYHA class III–IV on background conventional therapy), combining rhBNP (0.0075–0.01 μg/kg/min for 72 h) with ivabradine (titrated to achieve a resting heart rate of 55–60 bpm) for 1 month led to significantly greater improvements in LVEF, total effective rate, microcirculation, and neuroendocrine profiles than ivabradine alone (Zhang et al., 2026). Ivabradine reduces myocardial oxygen consumption by lowering heart rate, while rhBNP improves cardiac load by dilating blood vessels and promoting diuresis. Their mechanisms complement each other and each has its own advantages. They are suitable for patients with ischemic heart failure who have a fast heart rate.

### 5.3 Application strategies for different baseline BNP levels

The baseline BNP level of the patients is an important influencing factor for the efficacy of rhBNP and can be used as a biomarker to guide clinical medication. A research result shows that among patients with BNP ≤ 3,000 pg/mL, the effects of rhBNP in improving NYHA classification, reducing BNP level, and increasing LVEF are significantly better than those of dobutamine; however, in patients with BNP > 3,000 pg/mL, there is no significant difference in efficacy between the two (Pan et al., 2014). However, this 3,000 pg/mL cutoff should be interpreted cautiously as a hypothesis-generating observation that warrants further external validation, rather than an established clinical decision threshold. This phenomenon may be related to “natriuretic peptide resistance”. When the endogenous BNP is abnormally elevated, the targeted effect of exogenous rhBNP will be severely restricted. The molecular biological mechanism mainly involves the following key steps: (1) Receptor downregulation and saturation: The extremely high level of endogenous BNP is constantly exposed, which not only causes the natriuretic peptide receptor-A (NPR-A) on the cell membrane to be fully bound and reach saturation, making it difficult for exogenous drugs to effectively bind, but also triggers the endocytosis and degradation of the receptor, resulting in a significant decrease in the overall expression level of the receptor. (2) Receptor Desensitization: Under the downstream feedback caused by high-load ligand stimulation, the kinase homology domain (KHD) in the intracellular segment of NPR-A undergoes abnormal dephosphorylation modification, resulting in functional desensitization of the receptor. Even if the exogenous rhBNP binds successfully, the conversion efficiency of its catalyzing the conversion of GTP to the second messenger cGMP will significantly decline. (3) Compensation of degradation enzymes: Under severe heart failure conditions, the clearance receptors responsible for removing natriuretic peptides (NPR-C) and peripheral neuropeptidase (NEP) in the body may exhibit compensatory upregulation of expression, accelerating the degradation of exogenous rhBNP. Therefore, for patients with BNP ≤ 3,000 pg/mL, rhBNP is superior to dobutamine as a treatment option; for patients with BNP > 3,000 pg/mL, rhBNP has the same efficacy as dobutamine, and the choice can be made by considering factors such as drug adverse reactions and costs. In addition, the baseline BNP level can also be used to predict the therapeutic effect of rhBNP and guide individualized medication. Crucially, clinical evaluation of baseline BNP levels must account for key confounding variables. Circulating BNP concentrations are frequently elevated by advanced age, female sex, renal dysfunction, and atrial fibrillation, whereas obesity tends to suppress them. Additionally, the precise timing of blood sampling relative to acute symptom onset can cause substantial fluctuations. Integrating these factors is essential for accurate baseline risk stratification and clinical decision-making.

### 5.4 Application strategies for special groups

Older patients (aged >75 years) are more sensitive to the blood pressure-lowering effect of rhBNP due to decreased vascular compliance and reduced renal function. Therefore, a lower initial dose (0.005–0.01 μg/kg/min) should be used, and the titration interval should be extended to prevent hypotension and renal function damage (Blumer et al., 2021). In pediatric acute heart failure, evidence for rhBNP remains limited to pilot evaluations. A prospective study of 32 pediatric patients demonstrated that continuous rhBNP infusion without an initial bolus, starting at 0.01 μg/kg/min and titrated up to 0.03 μg/kg/min, was well tolerated alongside standard vasoactive therapy. Treatment significantly increased urine output and lowered central venous pressure without causing hypotension or renal impairment. Given the open-label design of current studies, randomized trials are still required to establish standardized pediatric protocols (Jefferies et al., 2006). In patients undergoing cardiac surgery with cardiopulmonary bypass (CPB), rhBNP is applied to attenuate perioperative organ injury. A retrospective study of 357 post-CPB patients demonstrated that continuous rhBNP infusion (0.0075–0.01 μg/kg/min for 72 h) significantly accelerated myocardial recovery with faster serum troponin clearance. It also conferred renal protection by increasing urine volume and attenuating serum creatinine elevation while lowering central venous pressure. However, rhBNP did not improve overall clinical prognosis, ICU stay duration, or in-hospital mortality, underscoring the necessity for prospective randomized trials (Le et al., 2017). For pregnant patients, there is a lack of safety data for rhBNP. It should be used with caution only when the benefits outweigh the risks, and fetal conditions need to be closely monitored. Cardiovascular diseases during pregnancy (such as peripartum cardiomyopathy) can endanger both the mother and the fetus. The use of rhBNP should be based on individualized assessment and managed in conjunction with a multidisciplinary team. Overall, the treatment of rhBNP for special populations should follow the principle of “low-dose initiation and individualized titration”, and enhanced monitoring should be implemented to balance efficacy and safety.

## 6 Conclusions and prospects

rhBNP mimics the physiological effects of endogenous BNP and exerts multi-target protective effects in cardiovascular diseases. In terms of hemodynamics, rhBNP can rapidly reduce the preload and afterload of the heart within 30 min of administration by balancing the dilation of arteries and veins as well as the coronary arteries. It also increases coronary perfusion and reduces myocardial oxygen consumption, and the effects can persist for up to 6 h after discontinuation of the drug. In terms of clinical treatment strategies, the low-dose rhBNP regimen is safe and effective. The baseline BNP level of patients can be used to guide the medication. It has a synergistic effect when combined with positive inotropic drugs, sacubitril/valsartan sodium, ivabradine, *etc.* For special populations, the principle of “low-dose initiation and individualized titration” should be followed.

Although existing studies have demonstrated that rhBNP has multi-target protective effects in acute cardiovascular diseases, most clinical studies are designed as combination therapies. The independent effect of rhBNP as a single drug has not been verified, and the current research sample size is small, and the long-term prognosis data is limited. In the future, large-scale, multicenter, double-blind, placebo-controlled trials should focus on hard, patient-centered endpoints (e.g., survival, rehospitalization, and quality of life) to confirm whether these short-term physiological improvements translate into long-term clinical benefits.
